# Adapting to Uncertainty: Foraging Strategies in *Dinoponera quadriceps* (Formicidae: Ponerinae)

**DOI:** 10.3390/insects15120948

**Published:** 2024-11-30

**Authors:** Igor Eloi, Waldemar Alves Silva-Neto, Wallisen Tadashi Hattori, Arrilton Araújo

**Affiliations:** 1 Laboratório de Biologia Comportamental, Departamento de Fisiologia e Comportamento, Universidade Federal do Rio Grande do Norte, Natal 59078-970, RN, Brazil; igor.eloi.108@ufrn.edu.br (I.E.); alvesneto@gmail.com (W.A.S.-N.); 2 Departamento de Saúde Coletiva, Faculdade de Medicina, Universidade Federal de Uberlândia, Uberlândia 38405-320, MG, Brazil; wallhattori@gmail.com

**Keywords:** patch residency time, solitary foraging, ants, foraging dynamics

## Abstract

When searching for food, animals often make decisions about where to go, how long to stay in a foraging area, and whether to return to the most recently visited spot. These decisions can be enhanced by cognitive traits and adjusted based on previous experience. In social insects, such as ants, foraging efficiency has an impact at both the individual and colony levels. The present study investigated the effect of the distance to, capture success, food size, and the reward rate on decisions of where to forage in *Dinoponera quadriceps*, a ponerine ant that forages solitarily and makes individual foraging decisions, in laboratory studies. We also investigated the influence of learning on the workers’ performance over successive trips to search for food by measuring the patch residence time in each foraging trip. Four scenarios were created that differed in the food reward rates, the food size offered, and the distances from the colony to the food site. Our work demonstrated that as a general rule, the *D. quadriceps* workers return to the place where a prey item was found on the previous trip, regardless of the distance, food size, and reward rate. When the ants did not capture prey, they were more likely to change their route to search for food. Our results also indicated a learning process for the routes of exploration, as well as the food site conditions for exploration. After repeated trips, the foragers reduced the patch residence time in areas where they did not capture food and quickly changed foraging areas, increasing their foraging efficiency.

## Simple Summary

We conducted an investigation into the foraging behavior of *Dinoponera quadriceps*, which is an ant species that forages solitarily. The ants were subjected to a range of foraging scenarios that included varying distances to food sources, differing prey sizes, and varying capture success rates. The findings revealed that these ants display a significant preference for returning to previously successful foraging sites, irrespective of the distance, prey size, or reward rate. Conversely, in instances where the ants were unsuccessful in capturing prey, they exhibited a heightened tendency to explore new foraging areas.

## 1. Introduction

Foraging is an essential activity for an animal’s survival. Animals seeking food in dynamic habitats must frequently select where to allocate their foraging effort among different places in order to optimize their fitness [1]. However, there is a trade-off between exploration and exploitation since increasing the time spent searching for a new resource decreases the time spent exploring a known food source [2]. The optimal allocation of time to explore and exploit diverse food locations has a strong influence on both the individual and collective levels in the case of social insects [3].

Broad cognitive abilities such as learning and memory may improve foraging efficiency [4,5]. Learning is the process of acquiring and retaining information, resulting in modifications in behavior based on past experiences, together with the ability to use the stored information (memory), it helps animals adapt to changing environments and situations [6]. For instance, improvements in efficiency during foraging can be achieved through adjusting the patch residency duration based on previously acquired information on the reward rate within the patch [7], or assuming alternating strategies (“win-stay” and “lose-shift”) based on the payoff from the previous experienced on the last trip [8]. Hence, patch residence time could be interpreted as an accumulation process where information from their previous experience is used to make informed decisions in the future [9], which can manifest in the investment of more searching to make up for efficiency deficits [10].

Models of optimal patch residence time assume that animals can assess and adapt to their foraging environment cognitively. Another strategy for increasing efficiency is to invest more in searching (i.e., energy and time).

Ants are eusocial insects with fascinating foraging strategies [11]. Some species, such as *Ectatomma ruidum* and *Paraponera clavata*, utilize both individual foraging and group recruitment or tandem-running [12,13,14,15], others, including *Neoponera apicalis* [16], *Cataglyphis bicolor* [17], *Hagensia havilandi* [18], and *Dinoponera quadriceps* [19], use a solitary strategy. Individuals leave the nest, search for food, capture and transport it without systematic cooperation or communication [20]. When an individual embarks on a foraging trip alone, it is forced to rely solely on egocentric information to explicitly make choices, but that can be adjusted based on previous experiences [21]. *Dinoponera quadriceps* workers are monomorphic solitary ground foragers that do not recruit workers to forage, they use individual markings to develop their own routes. During outbound searching, the worker’s route follows an idiosyncratic direction, but the trajectory is erratic, as to maximize their searching field. When an ant captures a prey (mainly arthropods and fruits [22,23]), it dashes back to its nesting borrow in a seemingly straight line [18,21]. The amount of time spent foraging is proportional to their previous bound’s success: failing in capturing will result in greater latency on the next trip [18]. Above all, it implies that the ants are able to register and integrate their prior performances into the decision on how much time must be budgeted for the upcoming trip.

In this study, we investigated individual foraging decisions of *D. quadriceps*, a ponerine ant that forages alone and has no known recruiting mechanism [22], rendering it a species fit for studying individual components of cognition (e.g., decision-making). We explored whether and how factors like capture success, prey size, distance, and reward rate influence the decision to return to a previously searched location. We were particularly interested in the foragers’ cognitive capacities and how it affected their foraging behavior. We evaluated whether *D. quadriceps* foragers can learn the possible status (i.e., presence or absence of prey and quality of prey) of reward sites after multiple visits by assessing their patch residence time. Our hypothesis was that foragers will determine whether to stay or leave a route on each consecutive foraging excursion, minimizing search effort and increasing foraging efficiency.

## 2. Methods

### 2.1. Studied Species and Rearing of Ant Colonies

We used four colonies of *Dinoponera quadriceps* collected at the surroundings of the UFRN’s campus, in Natal, Rio Grande do Norte, Brazil. Colonies “C”, “E”, “G”, and “H” contained, respectively, 15, 9, 30, and 28 workers (average 20 ± 5.3 standard error; the avg. colony size for the species is 55 workers [24])—all containing brood. The colonies were kept in plastic boxes (30 × 15 × 10 cm) within arenas built of wood and coated with formica (a commercial material used to decorate surfaces with wooden texture) in the laboratory (100 × 50 × 20 cm). All ants were labeled with a unique numbered plastic tag attached to the thorax with ester-based cyanoacrylate glue [25]. Within the lab, room temperature varied from 25 to 30 degrees Celsius, relative humidity was set at 70%, and there was a consistent photoperiod of 12 h of light every day.

### 2.2. Experimental Setup

**Figure 1:**
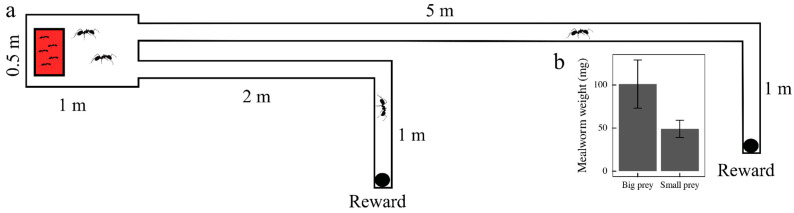
(**a**) The experimental setup is represented schematically. The ant nest (red rectangle) was housed in a wood arena where the ants were free to explore. At each round, a single forager was allowed into the route. The arms were tunnel-shaped and fashioned with plastic tubing (see-through hose), allowing the observer to watch the whole journey taken by a forager. (**b**) The average weight of the mealworms offered as rewards: Big Prey or Small Prey. Big Prey have roughly double the energy value offered by Small Prey.

The experimental setup (Figure 1) consisted of an arena (100 × 50 × 20 cm) connected to two tunnel-shaped plastic arms (a hose) (∅ = 90 mm) of 300 (short route) and 600 cm (double costly—long route) with blind end. *Dinoponera quadriceps* foragers routinely mark their trails with chemical signals that can be integrated for spatial orientation [26]. Here, we acknowledge the possibility that similar markings could have been made within the routes. Nevertheless, since we inserted a 24 h time lag between essays, we assume that the marking has, ipso facto, no considerable impact on the decision. At the end of each arm, we installed a Petri dish (∅ = 35 mm) in which we could place the reward (mealworm) through a hole in the hose’s ceiling (Figure 1a). Each arm and its, respectively, rewarding site were regarded as an independent route.

### 2.3. Experimental Procedure

Each day, we observed one forager. Other active workers which left the nesting arena during the experiment were removed and placed in a black box. In order to ensure the proper motivational state of the foragers [27,28], colonies were starved for two days before each day of observation. During the starvation period, we allowed all workers to freely roam the arms. To ensure familiarity with both routes, we only selected foragers who had visited both food spots at least once. Before conducting the tests, we categorized the mealworms based on their length and weight to determine their proportional rewarding value. The smaller mealworms (hereafter referred to as “small preys”)—size 10 ± 2 mm, weigh 49 ± 10 mg (mean ± SD), N = 26). In contrast, the larger mealworms hereafter referred to as “big preys”—size 21 ± 3 mm, weigh 101 ± 28 mg (mean ± SD), N = 31. Thus, one prey was considered to be twice as more valuable in terms of energy content than the other (Figure 1b).

We evaluated 21 foragers, each of which made 10 foraging visits in each (in total 40 visits per worker) of the following 4 experimental settings:

- Scenario 1: Upon arrival at a reward site, a forager encounters only one condition: a single big prey. The reward rate is 100%.
- Scenario 2: Upon arrival at a reward site, a forager encounters one of two possible conditions: either a single big prey item or no prey item. The reward rate is 50%.
- Scenario 3: Upon arrival at a reward site, a forager encounters one of two possible conditions: either a single small or a single big prey item—100% reward rate; 50% reward rate for each prey size.
- Scenario 4: Upon arrival at a reward site, a forager encounters one of three possible conditions: a single small prey item, a single big prey item or no prey item—66% reward rate; 33% reward rate for each prey size.

During each scenario, prey availability was haphazardly distributed among the ten excursions made by each forager. When the targeted individual was not on the route, mealworms were either replaced or removed. This ensured that foragers were not aware of any replacement until they reached the rewarding site. To minimize any potential influence of recent experience on a forager’s performance in subsequent scenarios, we ensured that we did not repeatedly test the same worker by manually controlling access to the arms.

Long-term memory in insects is a phenomenon that has been well-documented in scientific research. Overall, the storage of long-term memories in insects can be constrained from as little as 24 h [29,30,31], to as long as 6 months after acquisition, as seen in southern Finnish populations of the ant species *Formica uralensis* following winter diapause [32]. However, there is currently no information available (to the best of our understanding) on the long-term memory capabilities of *D. quadriceps* foragers. To account for this lack of knowledge, each forager in the study was given a one-month interval between trials of different scenarios. Additionally, the experimental design was specifically designed to feature only unidirectional routes, which allowed us to control for fidelity to a foraging area, as outlined by Araújo and Rodrigues [22].

To familiarize the ants with the experimental conditions, each forager was given a pre-experiment run on the maze with a 100% rewarding rate. Experimentation only began after the workers successfully captured the prey in the pre-trial run. In the subsequent ten trips, we recorded the following data: (1) the route selection (short or long route); (2) the round-trip time (time spent until finding the reward and time until returning to the nest after finding it); (3) whether or not the forager acquired a prey before returning to the nest; (4) the size of the prey; and (5) the decision made in the next trip (route fidelity or patch change). We coded the ant decision as a dummy variable, with the decision to “return” to the previously visited route coded as zero (0) and the decision to “change” routes coded as one (1). The time measurements were rounded to the nearest minute. After each day of observation, a forager made 10 decisions, resulting in 210 recorded decisions at the end of each scenario, and a total of 840 decisions at the completion of the study.

### 2.4. Statistical Analyses

All the data analyses were conducted using R version 4.4.2 [33]. Prior to formal analyses, we determined the most appropriate error distribution using the *fitdistrplus* package version 1.2-1 [34]. Following that, we adjusted a series of Generalized Linear Mixed Models (GLMM) using the *glmmTMB* package version 1.1.9 [35], with the error distribution based on the results of the *fitdistrplus* analysis. The selected error distribution was confirmed through residual inspection using the *DHARMa* package version 0.4.7 [36]. In order to investigate if the *D. quadriceps* foragers familiarized themselves with the experimental setting, we built a lognormal LMM using the time until finding the reward as a response of the trip number. The rationale behind this model is that as ants undergo trips, their performance improves, which would be observed through reduction in the time until finding the reward. The model incorporated fixed factors of scenarios and nests, with random intercepts for individuals to account for variations originating from individual performances. A post hoc pairwise comparison was conducted using the *emmeans* package version 1.10.5 [37] to detect performance differences among colonies. Because the model showed a significant interaction term, the emtrends function was used to account for the influence of trip order (1–10). To correct the comparisons for inflated statistical error, the *p* values were adjusted using the Bonferroni correction.

In order to test how the scenarios influence individual foraging decisions of either returning to the same route previously visited or looking for food in the other route (decision—0/1), we built GLMMs under Binomial error distribution. To simplify the presentation of findings, and to be able to explore the situations in depth, we fitted individual models for each scenario, hence assuming that the order of exposure of scenarios has no statistical influence over the performance of an ant in the subsequent scenario. Since we used a within-subject design (the same ants underwent all the conditions), we accounted for individual variability in foraging behavior, as well as spontaneous alternation behavior (SAB) [38], by including individuals and the order of the trip as random factors in the models. As our colonies varied in size, they were likely to have different foraging requirements that could affect foragers’ decisions. Therefore, we also included “colony” as a fixed term in the models, following Bolker’s [39] recommendation to treat factors with fewer than five levels as fixed terms to avoid low statistical power as random terms. The models were first built with the following explanatory variables: chosen route (long or short), capture (0 or 1), and colony (C, E, G, H). The *buildmer* package version 2.11 [40] was used to automate the model selection, basing the selection in the Akaike criteria. The simplification was set to drop fixed terms while keeping the random structure. Only the final models were considered in our results.

We used the *coxme* package version 2.2-22 [41] to fit a mixed-effects Cox model and examine how the time taken for foragers to return to the nest varied over the course of 10 trips and whether prey capture influenced this response. The model included fixed effects for the occurrence of the capture and experimental scenarios, as well as random effects for the ant and trip. We simplified the model by removing fixed terms and their interactions that did not contribute significantly (*p* > 0.05) to the fit. Whenever needed, estimated marginal means (EMM) were computed using the *emmeans* package [37]. All the models were tested using Analysis of Deviance with Wald chi-squared test.

## 3. Results

### 3.1. Performance over Time

**Figure 2:**
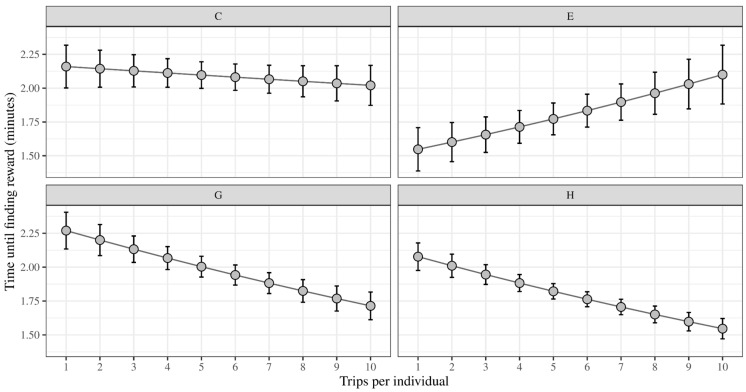
Performance curves: estimated marginal means (EMM) for the time until discovering the reward as a function of the number of visits made by each individual. The means are represented by the dots, while the ranges reflect the standard error of the means. The use of EMM allowed us to control for individual differences in performance, and hence provided a clearer picture of the colonial variation. Colony C shows a decrease in the reward finding delay, while E has increased the time. G and H have linearly decreased their time until finding reward.

Overall, we found no significant change in the time taken by *D. quadriceps* workers to locate the reward as they repeated their travels (lognormal GLMM-*χ*^2^ = 0.34; *p* = 0.555). Based on the marginal means of the model, overall the ants took an average of 1.99 (±0.16 SE) minutes on their first trip, which was reduced by 8.0% on their tenth trip. However, we did find a significant difference among colonies (lognormal GLMM-*χ*^2^ = 11.03; *p* = 0.011) and an interaction between trip and colony (lognormal GLMM-*χ*^2^ = 13.70; *p* = 0.003). During the experiment, we observed variation in the performance of different colonies. Colony C maintained a relatively constant level of performance across all ten trips, while colony E showed an increase in the time taken to find the reward. On the other hand, colonies G and H both showed a decrease in time, indicating an improvement in efficiency (Figure 2). For a more detailed comparison of the colonies, see pairwise contrasts in Table 1.

### 3.2. Route Decision Scenario 1

**Figure 3:**
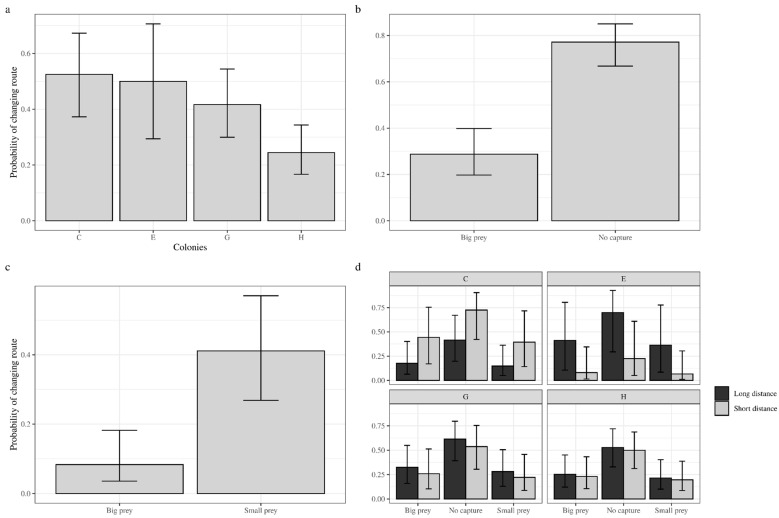
Marginal means and 95% confidence intervals for the mixed models representations of the four tested scenarios. (**a**) Scenario 1 (100% reward rate, big prey)—in a situation where reward is guaranteed, the foragers were more likely to change routes when they opted first to undergo the long distance route. Additionally, the probability of changing routes varied depending on the colony of origin, with colony H (28 workers) showing the smallest rate of route changing. (**b**) Scenario 2 (50% reward rate, big prey): since the capture was marginally significant, we included it as a factor. When capture was available, the probability of changing route is higher in the longer route. When no reward is offered, the probability of changing between both routes is subequal. (**c**) Scenario 3 (100% reward rate, ½ big or small prey): the foragers are more likely to change routes when the reward is small, but there is a marginally significant influence of route length. (**d**) Scenario 4 (66% reward rate, ⅓ none, big or small prey): the foragers are more likely to change routes when there is no capture. The probability of changing routes is alike for big and small prey, but colony C is more likely to change route if the shorter route was taken first.

When presented with 100.0% reward rate, the colonies were reported as the factor influencing the decision (binomial GLMM, *χ*^2^ = 11.793; *p* = 0.008) (Figure 3a). This finding suggests that when guaranteed reward is available, the nutritional requirements of the colony become a pivotal factor influencing the probability of foragers changing route. Specifically, the probability of changing routes was smaller in colony H.

### 3.3. Route Decision Scenario 2

When offered with a 50% reward rate, the previous trip’s capture is the key factor affecting the decision to return to that route (binomial GLMM, *χ*^2^ = 41.461; *p* < 0.001) (Figure 3b). Based on the marginal means of the model, when the previous trip did not result in a capture, the probability of changing routes increased 83.0% in the long route and 73.0% in the short route. During model simplification, the colony fixed term was dropped, suggesting that it had no statistical significance on the probability of changing route.

### 3.4. Route Decision Scenario 3

At 100.0% reward rate but different sizes (½ chance for either), the size of the offer is the most influential factor in the decision to return to that route (binomial GLMM, *χ*^2^ = 21.32; *p* < 0.001), with foragers 73.8% more likely to choose a different route if the previous trip rewarded a small prey (Figure 3c). Based on the estimated marginal means, this scenario reports that the probability of changing routes is 12.0% when big prey are found in the long route, and 52.0% when small prey is found. In contrast, in the short route, there is only a 6.0% chance of changing route when big prey is found and 34% when small prey is found. The fixed term for “colony” was dropped during model simplification, implying insufficient statistical relevance.

### 3.5. Route Decision Scenario 4

At 66.0% reward rate and ⅓ variation for reward rate, the foragers of *D. quadriceps* were more likely to change routes depending on the capture (binomial GLMM, *χ*^2^ = 15.09; *p* < 0.001). Nevertheless, the model reported a significant interaction term between the route and the colony (Z = −2.403; *p* = 0.016), with colony C (*n* = 15 workers) displaying higher probability of changing route if the shorter was firstly chosen. Failing to find capture resulted in a larger probability of route changing (53.0%), followed by big prey (25.0%) and small prey (22.0%) (Figure 3d).

### 3.6. Effect of Capture over Route Residency Time

**Figure 4:**
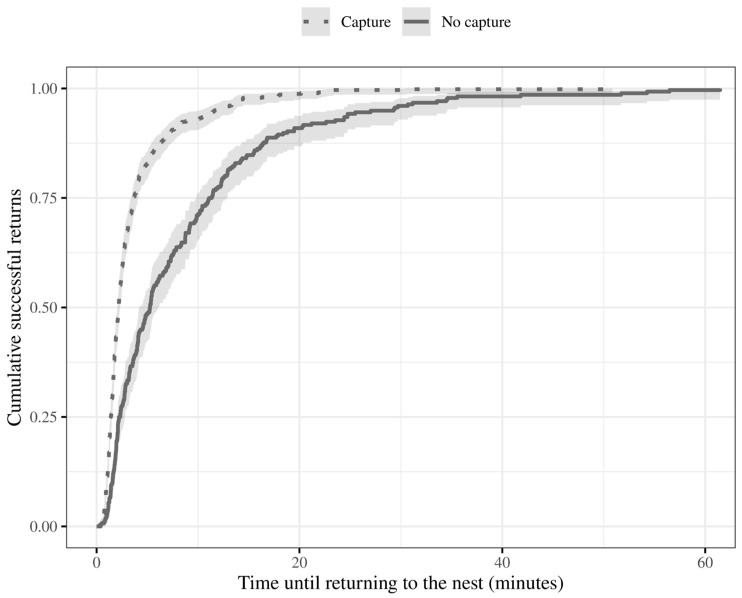
The foragers’ effectiveness under variable capture occurrence. The blue line depicts the accumulation of events where a forager was successful at capturing a reward. The blue line represents the accumulation of events where a forager was unsuccessful at capturing a reward. Shadows around the line represent the 95% confidence interval. Failing to capture is associated with workers staying longer on the routes.

The occurrence of capture significantly influenced the time taken to return from foraging (Cox GLMM *χ*^2^ = 129.77, *p* < 0.001), regardless of the scenario (the fixed term for scenarios was dropped during model simplification—*p* > 0.05). Specifically, ants that were more successful at capturing had a significantly shorter time until they returned to the nest compared to ants that were less successful (Figure 4). On average, successful captures spent 3.5 min (±0.18 SE) on the routes, while unsuccessful captures resulted in 8.5 min (±0.57 SE).

## 4. Discussion

We investigated how individual experience and contextual stochasticity in resources influence the foraging behavior of the ponerine *D. quadriceps* workers. Our findings reveal that the previous foraging excursion’s success has a strong influence on foragers’ decisions on the subsequent trip. In all scenarios, when ants collected either large or small prey, they were more likely to return to the previous trip’s route. Other factors, such as route length and reward size, had a modest influence on the decision. In addition, ants were able to maximize their efficiency throughout the essay.

Overall, we have not observed a conspicuous improvement in the performance of the studied ants on the routes; there was only an 8% reduction in the time taken to find the reward by the end of the experiments. However, the performance curves showed some variation depending on the colony, indicating colonial differences in learning [42]. Colony E increased the time until finding the reward, in a contrasting pattern to the decrease observed in all other colonies. Colony G and H have shown a clear decrease trend, which means their performance has actually improved over time. Since these colonies had the largest number of members, these results could suggest that the size of the colony might have an impact on the activity of their foragers [43].

A complementary explanation can be found in the phenomenon of colony profile [44,45], as evidence suggests that colonies with more active workers are prone to enter and explore new environments [46]. These findings can be integrated with the diffuse foraging behavior of *D. quadriceps* [47] to suggest that the non-cooperative foragers of this species may not heavily rely on individual learning, but colonies with quick learners (active workers) may gain an upper hand over competing neighbors as interspecific colony competition is a major part of their life-history [48].

In the scenario with a continuous reward (Scenario 1), we observed that some ants failed to capture prey and subsequently changed routes. Specifically, we observed that in 27% of visits, the ants did not capture prey but continued to explore the route. This phenomenon could be explained by SAB, which could often be prevalent over the within route stimuli when success is guaranteed. Our analysis revealed that the colony was the most significant factor in explaining the decision to change route in this scenario, indicating that the specific nutritional state of the colony, such as low resource demand [49] and protein satiation [50], might have influenced the decision-making of the foragers. Previous studies have shown various implications of food quality on ant foraging behavior. For instance, the searching effort and chosen route in *Formica pallidefulva* are heavily impacted by meal type. Workers were more persistent in foraging for carbohydrates than for protein food [51]. The desert ants *C. bicolor* persevered less in returning to a previously successful region if they had captured a fly in the previous excursion compared to times when they had gathered a slice of cheese [52]. Also, when provided sugar droplets, the formicine ants *Lasius niger* drank substantially more than when offered protein droplets [53].

In Scenario 2 (50% reward rate), the tendency to change routes after a failed trip suggests an influence of the absence of food on decision-making. In this case, it is possible to infer that ants have assumed a “loose-shift” strategy. Non-persistent foragers may have an advantage when looking for food that is unexpectedly dispersed [52], as was the case in Scenario 2. Short-term fluctuations in resource distribution are addressed by “sampling” the environment on a regular basis [54]. Indeed, *D. quadriceps* forages mostly for arthropods [22], a resource that is unpredictably distributed in the environment. While hunting area fidelity is a well-documented foraging strategy for ants [19,21], the direct attendance of individuals at a prior non-rewarding site has been seen only in rare and exceptional conditions. *Melophorus bagoti* foragers trained with a continual reward for two days returned to the same food location even after the feeder was withdrawn [55]. Moreover, Breed et al. [56] evaluated *P. clavata* workers in real settings and observed that after two consecutive incentives, some individuals (10 out of 21) returned to the feeder station several times more, even when no reward was offered. This is likely the case with *D. quadriceps* foragers in nature, since that happens when they failed to collect prey during the trials.

In Scenario 3, ants were once again exposed to a certain reward, but the value of the reward was variable. In this case, most foragers changed their route if a small prey was offered. The observed results suggest that workers might be able to individually evaluate each offer, store the information, and individually use it to decide whether they should return to that feeding site or explore a new area. This interpretation is supported by unpublished data that suggests that *D. quadriceps* foragers are able to integrate information regarding the weight and size of the resource and use it as part of their strategy (Azevedo & Araújo, unpublished). Invertebrates are likely to make decisions based on the perceived value of the reward [57,58,59], although there are also reports linking decisions to environmental cues and patch reward memory [60,61,62]. As all ants have been subjected to Scenarios 1 and 2 previously, it is not impossible that memory regarding prey size has been stored and accessed, even though a one-month interval was introduced. Another relevant observation is that the ants in Scenarios 1 and 3 (100% reward rate) differed in the number of visits without catching prey. The meal size supplied and how quickly colonies were satisfied during the studies might provide an explanation. In Scenario 1, the ants had big prey in all 210 excursions, but in Scenario 3, two different sizes of food were supplied at random. Whatever amount of energy a colony requires to continue its ecological operations may be obtained with fewer visits in Scenario 1 than in Scenario 3, resulting in a variable frequency of excursions in which the ants did not capture prey.

In Scenario 4, we increased complexity considerably and exposed ants to a mixed situation with uncertain rewarding rates. We also included uncertainty in the size of the reward, forcing ants to integrate the perceived reward value in their decision to change or stay in the chosen route. We observed that in such conditions, the primary factor that influenced ants’ decision to change route was the failure to capture a reward. Among the trialed scenarios, Scenario 4 exposed ants to what we could consider as the closest to general situations, making it the most representative of day-to-day scenarios. Another important interpretation is that ants may adapt their decision rate based on recent experiences, showing some level of behavioral flexibility to adjust their foraging strategy to respond better to prey availability. A similar use of previous experiences in adjusting decisions has been observed in various aspects of the ants’ life history both at colony [63,64], and individual level [65]. From an ecological point of view, this would be a valuable behavioral adaptation to better adjust their foraging in a changing environment where resource availability is diffusely spread. Indeed, the resources explored by *D. quadriceps* workers are stochastically distributed [22]. In our trials, the distance was less important in terms of the decisions made after each catch success (big prey, small prey, or no prey). There was no difference in foraging along the short and long routeways. The distances simulated in this study were less than the median distance traveled by *D. quadriceps* in their native environment (24.67 m) [19]. Although we did not find a significant influence of distance on *D. quadriceps* food seeking behavior, several studies have found this effect in other ant species [21]. To study any influence of distance, future laboratory work on this species should consider both longer distances and higher reward variety.

By integrating our observations, we concluded that the foragers of *D. quadriceps* are prone to a loose–shift foraging strategy, but their decision is at least partially informed by perceived value or perceived cost to capture a reward. As a confirmatory investigation, we predicted that if these ants really were loss-averse foragers, they might want to invest time into making sure to check for rewards before returning to the nest. Indeed, that was the case. Ants that failed to capture the reward spent more than double the time successful foragers did. The patch residency time was gradually reduced with repeated visits to the food locations, to the point where the time to depart a route following successful and failed journeys was statistically the same, showing that *D. quadriceps* had learned and memorized simple properties of the food sites. Foragers may adapt their time and energy allocation while looking for energy sources. Faster decisions mean less time and energy spent on unprofitable routes, and hence an increase in foraging efficiency. *Ectatomma* foragers boosted their foraging efficiency by making temporal and directional modifications depending on prior foraging success, according to Franz and Wcislo [66]. Moreover, Schwarz and Cheng [67] demonstrated that the Australian desert ant *M. bagoti* learned visual association tasks on their trip back to the nest in addition to discriminating visual stimuli connected with the reward.

In conclusion, we established in this study that the success of prior foraging expeditions is one key factor driving *D. quadriceps* food search decisions, suggesting that these ants favor a win–stay foraging strategy. In general, ants tend to return to the site of prior success and begin a new search from there when they are unsuccessful. We also demonstrated the role of cognitive features in improving foraging efficiency by reducing foraging efforts on unproductive routeways.

## Figures and Tables

**Table 1:** Pairwise comparisons of the time until finding reward among colonies, using the estimated marginal means. The contrast assumed a significance level of 0.05, and *p*-values were corrected using the Bonferroni adjustment. Significant differences are bolded.

Comparison	Estimated Contrast	T-Value	*p*-Value
C-E	−0.04131	−1.899	0.3476
C-G	0.02379	1.467	0.8566
C-H	0.02542	1.684	0.5551
E-G	0.06510	3.174	**0.0094**
E-H	0.06673	3.398	**0.0043**
G-H	0.00164	0.124	1

## Data Availability

The spreadsheet used for analyses is available as Supplementary Materials.

## Supplementary Materials

The following supporting information can be downloaded at: https://www.mdpi.com/article/10.3390/insects15120948/s1, Table S1.

## References

[1] Pyke G.H. (1984). Optimal Foraging Theory: A Critical Review. Annu. Rev. Ecol. Syst..

[2] Latty T., Beekman M. (2013). Keeping Track of Changes: The Performance of Ant Colonies in Dynamic Environments. Anim. Behav..

[3] Cerdá X., Angulo E., Boulay R., Lenoir A. (2009). Individual and Collective Foraging Decisions: A Field Study of Worker Recruitment in the Gypsy Ant *Aphaenogaster senilis*. Behav. Ecol. Sociobiol..

[4] McNamara J.M., Houston A.I. (1985). Optimal Foraging and Learning. J. Theor. Biol..

[5] Czaczkes T.J. (2022). Advanced Cognition in Ants. Myrmecol. News.

[6] Johnson R.A. (1991). Learning, Memory, and Foraging Efficiency in Two Species of Desert Seed-Harvester Ants. Ecology.

[7] McNair J.N. (1982). Optimal Giving-up Times and the Marginal Value Theorem. Am. Nat..

[8] Nowak M.A., Sigmund K. (1994). The Alternating Prisoner’s Dilemma. J. Theor. Biol..

[9] Davidson J., Hady A. (2018). Foraging as an Evidence Accumulation Process. PLoS Comput. Biol..

[10] Fewell J. (1988). Energetic and Time Costs of Foraging in Harvester Ants, *Pogonomyrmex occidentalis*. Behav. Ecol. Sociobiol..

[11] Hölldobler B., Wilson E.O. (1990). The Ants.

[12] Levings S.C., Franks N.R. (1982). Patterns of Nested Dispersion in a Tropical Ground Ant Community. Ecology.

[13] Breed M.D., Fewell J.H., Moore A.J., Williams K.R. (1987). Graded Recruitment in a Ponerine Ant. Behav. Ecol. Sociobiol..

[14] Fewell J., Harrison J., Stiller T., Breed M. (1992). A Cost–Benefit Analysis of Distance Effects on Foraging and Recruitment in the Giant Tropical Ant, *Paraponera clavata*. Oecologia.

[15] Schatz B., Lachaud J.-P., Beugnon G. (1996). Polyethism within Hunters of the Ponerine Ant, *Ectatomma Ruidum* Roger (Formicidae, Ponerinae). Insectes Sociaux.

[16] Fresneau D. (1985). Individual Foraging and Path Fidelity in a Ponerine Ant. Insectes Sociaux.

[17] Wehner R., Harkness R.D., Schmid-Hempel P., Lindauer M. (1983). Foraging Strategies in Individually Searching Ants, *Cataglyphis Bicolor* (Hymenoptera: Formicidae). Akademieder Wissenschaften und der Literatur, Mainz, Mathematisch-Naturwissenschaftliche Klasse.

[18] Duncan F., Crewe R. (1994). Field Study on the Foraging Characteristics of a Ponerine Ant, *Hagensia havilandi* Forel. Insectes Sociaux.

[19] Azevedo D.L.O., Medeiros J.C., Araújo A. (2014). Adjustments in the Time, Distance and Direction of Foraging in *Dinoponera quadriceps* Workers. J. Insect. Behav..

[20] Beckers R., Goss S., Deneubourg J.-L., Pasteels J.-M. (1989). Colony Size, Communication and Ant Foraging Strategy. Psyche.

[21] Traniello J.F.A. (1989). Foraging Strategies of Ants. Annu. Rev. Entomol..

[22] Araújo A., Rodrigues Z. (2006). Foraging Behavior of the Queenless Ant *Dinoponera quadriceps* Santschi (Hymenoptera: Formicidae). Neotrop. Entomol..

[23] Medeiros J., Azevedo D.L.O., Santana M.A.D., Lopes T.R.P., Araujo A. (2014). Foraging Activity Rhythms of *Dinoponera quadriceps* (Hymenoptera: Formicidae) in Its Natural Environment. J. Insect Sci..

[24] Vasconcellos A., Santana G.G., Souza A.K. (2004). Nest Spacing and Architecture, and Swarming of Males of *Dinoponera quadriceps* (Hymenoptera, Formicidae) in a Remnant of the Atlantic Forest in Northeast Brazil. Braz. J. Biol..

[25] Corbara B., Fresneau D., Lachaud J.-P., Leclerc Y., Goodall G. (1986). An Automated Photographic Technique for Behavioural Investigations of Social Insects. Behav. Process..

[26] Azevedo D.L.O., Santos P.F., Pereira A.G.C., Corso G., Araújo A. (2022). Effect of Chemical and Visual Cues in the Maze Performance of the Ant *Dinoponera quadriceps*. J. Insect Behav..

[27] Josens R.B., Roces F. (2000). Foraging in the Ant *Camponotus mus*: Nectar-Intake Rate and Crop Filling Depend on Colony Starvation. J. Insect Physiol..

[28] Mailleux A.-C., Devigne C., Deneubourg J.-L., Detrain C. (2010). Impact of Starvation on *Lasius niger*’ Exploration. Ethology.

[29] Dreier S., Van Zweden J.S., D’Ettorre P. (2007). Long-Term Memory of Individual Identity in Ant Queens. Biol. Lett..

[30] Haehnel M., Menzel R. (2012). Long-Term Memory and Response Generalization in Mushroom Body Extrinsic Neurons in the Honeybee *Apis mellifera*. J. Exp. Biol..

[31] Menzel R. (2012). The Honeybee as a Model for Understanding the Basis of Cognition. Nat. Rev. Neurosci..

[32] Salo O., Rosengren R. (2001). Memory of Location and Site Recognition in the Ant *Formica uralensis* (Hymenoptera: Formicidae). Ethology.

[33] (2024). R Core Team R: A Language and Environment for Statistical Computing.

[34] Delignette-Muller M.L., Dutang C. (2015). Fitdistrplus: An R Package for Fitting Distributions. J. Stat. Softw..

[35] Brooks M.E., Kristensen K., van Benthem K.J., Magnusson A., Berg C.W., Nielsen A., Skaug H.J., Maechler M., Bolker B.M. (2017). glmmTMB Balances Speed and Flexibility Among Packages for Zero-Inflated Generalized Linear Mixed Modeling. R J..

[36] Hartig F., Lohse L., de leite M.S. DHARMa: Residual Diagnostics for Hierarchical (Multi-Level/Mixed) Regression Models. https://cran.r-project.org/web/packages/DHARMa/.

[37] Lenth R.V., Banfai B., Bolker B., Buerkner P., Giné-Vázquez I., Herve M., Jung M., Love J., Miguez F., Piaskowski J. Emmeans: Estimated Marginal Means, Aka Least-Squares Means. https://cran.r-project.org/web/packages/emmeans/index.html.

[38] Richman C.L., Dember W.N., Kim P. (1986). Spontaneous Alternation Behavior in Animals: A Review. Curr. Psychol. Res. Rev..

[39] Bolker B.M., Fox G.A., Negrete-Yankelevich S., Sosa V.J. (2015). Linear and Generalized Linear Mixed Models. Ecological Statistics: Contemporary theory and Application.

[40] Voeten C.C. Buildmer: Stepwise Elimination and Term Reordering for Mixed-Effects Regression Available online:. https://cran.r-project.org/web/packages/buildmer/index.html.

[41] Therneau T.M. Coxme: Mixed Effects Cox Models Available online:. https://cran.r-project.org/web/packages/coxme/index.html.

[42] Chittka L., Rossiter S.J., Skorupski P., Fernando C. (2012). What Is Comparable in Comparative Cognition?. Philos. Trans. R. Soc. B Biol. Sci..

[43] Nobua-Behrmann B.E., Lopez de Casenave J., Milesi F.A., Pavan B. (2013). Forager Abundance and Its Relationship with Colony Activity Level in Three Species of South American *Pogonomyrmex* Harvester Ants. Insect. Soc..

[44] Wray M.K., Mattila H.R., Seeley T.D. (2011). Collective Personalities in Honeybee Colonies Are Linked to Colony Fitness. Anim. Behav..

[45] Pinter-Wollman N. (2012). Personality in Social Insects: How Does Worker Personality Determine Colony Personality?. Curr. Zool..

[46] Pasquier G., Grüter C. (2016). Individual Learning Performance and Exploratory Activity Are Linked to Colony Foraging Success in a Mass-Recruiting Ant. Behav. Ecol..

[47] Araújo A., Câmara J., Azevedo D., Medeiros I., Neto W., Garcia D., Delabie J.H.C., Feitosa R.M., Serrão J.E., Mariano C.d.S.F., Majer J.D. (2015). Poneromorfas Sem Rainhas—*Dinoponera*: Aspectos Ecológico-Comportamentais. As Formigas Poneromorfas do Brasil.

[48] Medeiros J., Araújo A. (2014). Workers’ Extra-Nest Behavioral Changes during Colony Fission in *Dinoponera quadriceps* (Santschi). Neotrop. Entomol..

[49] Cassill D. (2003). Rules of Supply and Demand Regulate Recruitment to Food in an Ant Society. Behav. Ecol. Sociobiol..

[50] Csata E., Gautrais J., Bach A., Blanchet J., Ferrante J., Fournier F., Lévesque T., Simpson S.J., Dussutour A. (2020). Ant Foragers Compensate for the Nutritional Deficiencies in the Colony. Curr. Biol..

[51] Fourcassié V., Traniello J.F. (1994). Food Searching Behaviour in the Ant *Formica schaufussi* (Hymenoptera, Formicidae): Response of Naive Foragers to Protein and Carbohydrate Food. Anim. Behav..

[52] Schmid-Hempel P. (1984). Individually Different Foraging Methods in the Desert Ant *Cataglyphis bicolor* (Hymenoptera, Formicidae). Behav. Ecol. Sociobiol..

[53] Portha S., Deneubourg J.-L., Detrain C. (2004). How Food Type and Brood Influence Foraging Decisions of *Lasius niger* Scouts. Anim. Behav..

[54] Stephens D.W., Krebs J.R. (1986). Foraging Theory.

[55] Schultheiss P., Cheng K. (2013). Finding Food: Outbound Searching Behavior in the Australian Desert Ant *Melophorus bagoti*. Behav. Ecol..

[56] Breed M.D., Stierstorfer C., Furness E.D., Jeral J.M., Fewell J.H. (1996). Individual Constancy of Local Search Strategies in the Giant Tropical Ant, *Paraponera clavata* (Hymenoptera: Formicidae). J. Insect Behav..

[57] Persons M.H., Uetz G.W. (1996). The Influence of Sensory Information on Patch Residence Time in Wolf Spiders (Araneae: Lycosidae). Anim. Behav..

[58] Oberhauser F.B., Czaczkes T.J. (2018). Tasting the Unexpected: Disconfirmation of Expectations Leads to Lower Perceived Food Value in an Invertebrate. Biol. Lett..

[59] Oberhauser F.B., Schlemm A., Wendt S., Czaczkes T.J. (2019). Private Information Conflict: Lasius Niger Ants Prefer Olfactory Cues to Route Memory. Anim. Cogn..

[60] Louâpre P., van Baaren J., Pierre J.S., van Alphen J.J.M. (2011). Information Gleaned and Former Patch Quality Determine Foraging Behavior of Parasitic Wasps. Behav. Ecol..

[61] Louâpre P., Pierre J.-S. (2014). Parasitoids Update the Habitat Profitability by Adjusting the Kairomone Responsiveness to Their Oviposition Experience. Ecol. Entomol..

[62] Sheng S., Feng S., Meng L., Li B. (2014). Departure Mechanisms for Host Search on High-Density Patches by the *Meteorus pulchricornis*. J. Insect Sci..

[63] Stroeymeyt N., Robinson E.J.H., Hogan P.M., Marshall J.A.R., Giurfa M., Franks N.R. (2011). Experience-Dependent Flexibility in Collective Decision Making by House-Hunting Ants. Behav. Ecol..

[64] Sasaki T., Pratt S.C. (2013). Ants Learn to Rely on More Informative Attributes during Decision-Making. Biol. Lett..

[65] Grüter C., Czaczkes T.J., Ratnieks F.L.W. (2011). Decision Making in Ant Foragers (*Lasius niger*) Facing Conflicting Private and Social Information. Behav. Ecol. Sociobiol..

[66] Franz N.M., Wcislo W.T. (2003). Foraging Behavior in Two Species of *Ectatomma* (Formicidae: Ponerinae): Individual Learning of Orientation and Timing. J. Insect Behav..

[67] Schwarz S., Cheng K. (2011). Visual Discrimination, Sequential Learning and Memory Retrieval in the Australian Desert Ant *Melophorus bagoti*. Anim. Cogn..

